# The Honey Bee *Apis mellifera*: An Insect at the Interface between Human and Ecosystem Health

**DOI:** 10.3390/biology11020233

**Published:** 2022-02-01

**Authors:** Giulia Papa, Roberto Maier, Alessandra Durazzo, Massimo Lucarini, Ioannis K. Karabagias, Manuela Plutino, Elisa Bianchetto, Rita Aromolo, Giuseppe Pignatti, Andrea Ambrogio, Marco Pellecchia, Ilaria Negri

**Affiliations:** 1 Department of Sustainable Crop Production–DIPROVES, Università Cattolica del Sacro Cuore, Via Emilia Parmense 84, 29122 Piacenza, Italy; giulia.papa@unicatt.it; 2 Istituto per la Protezione Sostenibile Delle Piante, Consiglio Nazionale Delle Ricerche, IPSP-CNR, Strada delle Cacce 73, 10135 Torino, Italy; 3 Faculty of Agriculture, Food and Environmental Sciences, Università Cattolica del Sacro Cuore, Via Emilia Parmense 84, 29122 Piacenza, Italy; roberto.maier@unicatt.it; 4 CREA-Research Centre for Food and Nutrition, Via Ardeatina 546, 00178 Rome, Italy; massimo.lucarini@crea.gov.it; 5 Department of Food Science and Technology, School of Agricultural Sciences, University of Patras, Charilaou Trikoupi 2, 30100 Agrinio, Greece; ikaraba@upatras.gr; 6 CREA-Research Centre for Forestry and Wood, Viale Santa Margherita 80, 52100 Arezzo, Italy; manuela.plutino@crea.gov.it; 7 CREA-Research Centre for Agriculture and Environment, via di Lanciola 12/A, 50125 Firenze, Italy; elisa.bianchetto@crea.gov.it; 8 CREA-Research Centre for Agriculture and Environment, via Della Navicella 4, 00184 Rome, Italy; rita.aromolo@crea.gov.it; 9 CREA-Research Centre for Forestry and Wood, 00166 Roma, Italy; giuseppe.pignatti@crea.gov.it; 10 DRANAE, Via Borghetto 27, 29121 Piacenza, Italy; info@dranae.it; 11 KOINE’–Consulenze Ambientali, 43022 Montechiarugolo, Italy; marcopellecchia@koineambiente.com

**Keywords:** *Apis mellifera*, honey bee, ecosystem services, agro-ecosystems, bee products, provisioning services, regulating services, cultural services, biodiversity

## Abstract

**Simple Summary:**

*Apis mellifera* Linnaeus (1758), a honey bee, is a eusocial insect widely known for its role in pollination, an essential ecosystem service for plant biodiversity, and quality of vegetables and fruit products. In addition, honey bees and bee products are valuable bioindicators of pollutants, such as airborne particulate matter, heavy metals, and pesticides. In this review, we explore the provisioning, regulating, and cultural services provided by the honey bee, an insect at the interface between human and ecosystem health.

**Abstract:**

The concept of ecosystem services is widely understood as the services and benefits thatecosystems provide to humans, and they have been categorised into provisioning, regulating, supporting, and cultural services. This article aims to provide an updated overview of the benefits that the honey bee *Apis mellifera* provides to humans as well as ecosystems. We revised the role of honey bees as pollinators in natural ecosystems to preserve and restore the local biodiversity of wild plants; in agro-ecosystems, this species is widely used to enhance crop yield and quality, meeting the increasing food demand. Beekeeping activity provides humans not only with high-quality food but also with substances used as raw materials and in pharmaceuticals, and in polluted areas, bees convey valuable information on the environmental presence of pollutants and their impact on human and ecosystem health. Finally, the role of the honey bee in symbolic tradition, mysticism, and the cultural values of the bee habitats are also presented. Overall, we suggest that the symbolic value of the honey bee is the most important role played by this insect species, as it may help revitalise and strengthen the intimate and reciprocal relationship between humans and the natural world, avoiding the inaccuracy of considering the ecosystems as mere providers of services to humans.

## 1. Introduction

Ecosystems support human life by providing a number of services and benefits that have been categorised into provisioning (e.g., food, water, and raw materials), regulating (e.g., processes that regulate climate, floods, diseases, and pollination), and cultural services (e.g., recreation, tourism, wellbeing, inspiration, and mysticism) [1]. Services and benefits provided by ecosystems are also influenced by the supporting services, that is, services and processes that yield an indirect benefit to humans, such as nutrient cycling, soil formation, habitat provision, and biodiversity maintenance [1]. However, despite its usefulness in bringing the interest of economists, policymakers, and the public on environmental issues, considering ecosystems as mere providers of benefits and services to humans is overly simplistic, and the (mis-)use of the concept ‘Ecosystem Services’ may risk the devaluation of the role of nature and of humans themselves, and their intimate reciprocal relationship.

This manuscript aims to provide an updated overview of the benefits that the honey bee *Apis mellifera* Linnaeus (1758) provides to humans as well as ecosystems. First, we revised the role of the honey bee as a pollinator in natural environments, which may help to preserve and restore the biodiversity of wild plants. On the other hand, pollination in agro-ecosystems may enhance crop yield and quality, meeting the increasing food demand. We also highlighted the importance of beekeeping, a high-valued and income-generating activity, which provides humans with honey as high-quality food as well as substances used as raw materials and in pharmaceuticals. 

In addition, we propose the role of honey bees and their products as bioindicators of environmental pollution as a further ‘service’ provided by these insects to safeguard human and ecosystem health. 

Finally, the role of the honey bee in symbolic tradition, mysticism, and the cultural values of the bees’ habitats are also discussed. Overall, we suggest that the symbolic value of the honey bee is possibly the most important role played by this insect species, as it may help revitalise and strengthen the intimate and reciprocal relationship between humans and the natural world, avoiding the inaccuracy of considering the ecosystems as mere providers of services to humans.

## 2. Regulating Services: The Conservation of Plant Biodiversity and Enhancement of Crop Production

Pollination by insects is one of the services that ecosystems provide for free. Insect-driven pollination involves hundreds of plant species that are visited by insects to search for nectar and/or pollen. Indeed, while foraging, nectar- and pollen-feeding insects can unintentionally transfer pollen grains to the flower stigma, facilitating fertilisation. Even if the vast majority of animal-pollinated plants rely on insects, especially bees, nectar-feeding vertebrates, such as some mammal and bird species, can promote cross-pollination [2]. 

As pollination and plant–pollinator interactions are fundamental for the reproductive success and fruit production of flowering plants, this ecosystem service supports the maintenance of plant biodiversity and is strictly linked to all the supporting, regulating, and provisioning services that stem from terrestrial vegetation.

In the following paragraphs, an overview of the role of managed bees and in particular the species *A. mellifera* in the conservation of plant biodiversity and enhancement of crop yield and quality is provided.

### 2.1. Plant Biodiversity Conservation

Plant–pollinator relationships represent one of the most important drivers of biodiversity on Earth [3]: without pollinators, pollen and seeds cannot be transported and the reproduction of flowering plants would not be possible [4]. Pollination is not only directly responsible for the maintenance and diffusion of flowering species, but also supports the subsistence of other ecosystem components that depend on floral resources, such as herbivores and seed-eaters [5]. Flowers represent key microhabitats for a range of invertebrates and support a complementary fauna to leaves. Flowers are inhabited and visited by many species of micro- and macro-invertebrates for pollination rewards and floral herbivory, or because flowers act as resting/nesting sites or aggregation sites for mating or for predators [6]. Flowering plants are also believed to have promoted the radiation of diverse vertebrate and invertebrate animal lineages and epiphytic plants in tropical rainforests [7]. Given their exceptionally high transpiration capacities, angiosperms also play a key role in micro- and macro-climates, contributing to wet climate and precipitation, which are the main drivers of tropical biodiversity [7]. 

Wild and domesticated bees are the most important pollinator group and the role played by bees as pollinators within natural and agro-ecosystems is becoming increasingly evident and recognised [4,8,9].

While the importance of *A. mellifera* for crop production is widely acknowledged, there is currently a great debate among researchers on the real benefits to natural ecosystems derived from the presence of managed bees. Managed bee colonies may endanger wild pollinators, including wild populations of *A. mellifera* itself, due to floral resource limitation and potential pest and pathogen transmission [10,11]. This is especially true in the case of massive introduction of non-native honey bees in natural, protected areas [10,12]; therefore, according to some researchers, the best option should be to avoid high-density beekeeping and to increase spacing among neighbouring apiaries to guarantee abundant floral resources for all pollinators [10,13]. In view of this, laws and regulations to ban “intensive beekeeping” in natural ecosystems, while, in any case, favouring more sustainable approaches also through financial incentives for beekeepers should be promoted.

On the other hand, studies point out the global importance of honey bees as pollinators in natural habitats and the need to ensure their conservation to maintain the genetic diversity of local subspecies and their ecological function [11,14,15]. In natural habitats, honey bees appear to be the most frequent pollinators, averaging 13% of floral visits, with 5% of plant species being exclusively visited by *A. mellifera* [15]. This confirms that honey bees may also aid in the maintenance of the biodiversity of native communities of flowering plants [14,15,16].

Another important aspect linking *A. mellifera* and biodiversity is the possibility of using honey bees to understand the diversity status of flowering plants. Pollen richness is often used as a means to estimate the floristic richness of an ecosystem [17] and honey bees may provide useful information for monitoring purposes through, for example, analysis of the pollen grains packed into the pollen basket, as well as the analysis of pollen-contaminating bee products, especially honey. The use of molecular tools may offer further advantages in terms of quality and quantity of information compared to pollen identification through microscopic analysis. For example, DNA metabarcoding applied to honey reveals the presence of DNA from both pollen- and nectar-providing plants [18,19].

Of course, microscopic analysis and DNA metabarcoding applied to bee pollen and honey provide information on plant taxa on which honey bees forage, but, given that pollen composition in bee matrices is largely influenced by floristic local biodiversity and flowering phenology [18,19,20,21,22,23], these data may improve our understanding of the local biodiversity of flowering plants.

### 2.2. Crop Pollination: Quality and Yield

Pollination is a regulating service, with animal pollination playing a key role in the sexual reproduction of many crops—35% of global crop production—and wild plants [24]. 

Insect pollinators provide pollination services to over 70% of the world’s crops [4] and in the second half of the last century, they helped increase global food production between 15% and 30% with their free regulation service [25,26,27].

Eighty percent of global agricultural pollination services are attributed to *A. mellifera* [28], the most economically valuable pollinator of several crop monocultures worldwide. 

*A. mellifera* is easy to manage and transport, and the income the honey bee provides through the delivery of many products has made it the most valuable pollinator used to enhance agricultural production since ancient times. 

However, even if the honey bee is typically considered the most important pollinator, pollination also depends on wild bees and wasps (Table 1) [24,29].

In addition to Hymenoptera, insects belonging to other orders, such as Diptera, Coleoptera, and Lepidoptera, can provide pollination services similar to bees and, for some crops, they can also be highly efficient pollinators [30]. Diptera and Lepidoptera appear as the second and third most important orders of pollinating insects, visiting 72% and 54% of crops, respectively. Among the Diptera, hoverflies are recognised as important pollinators of wildflowers in many ecosystems, and their role as key pollinators of crops in vulnerable habitats, is attracting the interest of researchers [31]. 

In addition, some bee species promote pollination more efficiently than the honey bee: a typical example is the tomato (*Solanum lycopersicum*), a plant efficiently pollinated by bees that exploit vibrations to remove pollen from the flowers. In this case, the honey bee, which is incapable of vibrating flowers, displays reduced effectiveness [32]. As the scope of this review is to focus on the managed *A. mellifera* species, studies on the competition in gaining floral resources among honey bees and other pollinators or on positive associations that may arise between wild and managed species are not included. 

**Figure 1:**
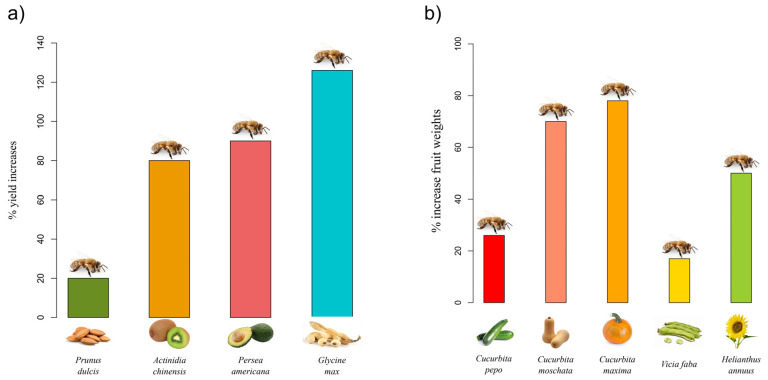
Benefits provided by *A. mellifera* in crop production. (**a**) Yield increase in almond, kiwi, avocado, and soybean crops; (**b**) increase in fruit weights in *Cucurbita* spp., fava bean, and sunflower.

Figure 1 provides an overview of the yield and weight increase in some crop products due to honey bee supplementation.

In large commercial orchards, insect pollination is typically enhanced to obtain marketable products and achieve high yields. One of the best examples is almond pollination in California, where more than 70% of all honey bee colonies in the USA are moved to orchards for the promotion of pollination. To overcome the increasing demand for managed bees, growers are starting to breed almond varieties claimed as ‘pollinator-independent’ due to their presumed great capacity for self-pollination [33]. However, researchers have found that even in this case if bees are not present in the orchards, growers obtain a lower crop yield, as pollinators guarantee a 20% increase in kernel yield (Figure 1a) [33].

In kiwi and avocado, while anemophilous and/or self-pollination alone ensures 12% and up to 17% of fruit set, respectively, when flowers are also exposed to honey bees, the yield increases to 80% and 90% (Figure 1a) [34,35]. In apples, one of the most important fruit crops in the world, insect pollination is necessary to obtain marketable fruits, i.e., large and symmetric [36]. Symmetry is a classical aesthetic principle, and consumers usually prefer to opt for beautiful fruits because they evoke naturalness and seem more appetising and healthier as compared to asymmetric fruits [37]. Therefore, honey bee colonies are usually placed into large commercial orchards to ensure fruit quality and quantity, even if honey bees do not seem to be the most efficient pollinators of apple flowers [38,39].

Similarly, supplementing raspberry and blueberry crops with beehives is necessary to obtain marketable berries [40,41]. Indeed, raspberry and blueberry fruits are characterised by an aggregation of drupelets, and under-pollinated flowers develop into crumbly, misshapen, or small berries that are avoided by consumers.

Honey bee supplementation is also important for ensuring yield stability over space and time [42]. If growers place small apiaries throughout a farm, this may enhance the spatial stability of bee visits, ensuring a homogeneous rate of yield quality and quantity [43]. The presence of healthy colonies also guarantees fruit quality and quantity across seasons in both apple and pear crops [44]. The strength of the bee colonies is also decisive for promoting crop production in the northern highbush blueberry, which is self-fertile, but higher fruit set and yields occur following visitation by honey bees from healthy colonies [45]. For watermelon crops, as native solitary bees are effective pollinators but do not allow optimal yield, supplementary pollination services through *A. mellifera* are suggested, even if in this case native managed stingless bees are preferable because they compete less with native pollinators [46]. In wild blueberry, both honey bee and bumblebee abundance increases fruit set and reduces spatial heterogeneity in crop production [47]. 

Honey bee supplementation is also known to improve yields in horticultural, legume, oilseed, and feed crops. In a recent study by Garibaldi et al. [48], the authors highlighted the fact that soybean productivity can significantly increase through insect pollination. Among soybean pollinators, it has been demonstrated that the honey bee effectively increases crop yield, pod set, and seed set [48]. In Brazil, crop yield has increased by up to 126% [48]. Honey bee supplementation enhances fruit weights of *Cucurbita pepo*, *C. moschata*, and *C. maxima* by approximately 26%, 70%, and 78%, respectively [49]; in fava bean (*Vicia faba*), the yield increased by 17% [50], and in sunflower (*Helianthus annuus*), about 50% [51] (Figure 1b). 

The effects of honey bees on fruit set and fruit/seed weight of biofuel crops, such as *Jatropha curcas* and *Ricinus communis*, have also been demonstrated [52,53]: *J. curcas*, for example, fruit set can increase up to 70% [53].

## 3. Use of Bee Products as Raw Materials and Medicinal Resources

Even if pollination is not unique to the honey bee, the delivery of a wide range of products to humans is exclusive to this insect. This has led to the development of beekeeping, a high-valued and income-generating activity, especially for honey production. In Europe the estimated number of hobby and professional beekeepers in 2010 was about 620,000, both with about 18.9 million hives and estimated honey production of more than 22,0000 tons [54]. Depending on the European country and distribution network, the price of honey can vary from a few euros to up to 40 euros/kg [54]. However, honey bees can provide not only food but also medicinal resources and raw materials.

Here, we review the literature on the importance of bee wax as a raw material, including new prospects for its use in a wide variety of industries, and the use of propolis, royal jelly, and venom in the pharmacological industry. This review does not include honey and pollen as medicinal resources, as they are treated in detail elsewhere [55,56,57,58].

### 3.1. Wax as Raw Material: New Perspectives

Beeswax is a secretion that adult bees aged between 12 and 18 days can produce from wax glands located in the abdomen. Once secreted, wax droplets solidify and are manipulated by the bee to build the nest, allowing food storage, brood rearing, and thermoregulation [59]. Beeswax is mainly composed of alkanes, fatty acids, long-chain esters, and trace compounds, including proteins and fragments of insects, plants, propolis, and pollen [60,61].

The use of beeswax by humans traces back to the Palaeolithic Age when early humans began to produce weapons for hunting by fixing stone tips to wooden shafts with a glue substance made of beeswax and resins [62]. Hunting was also enhanced by using poisonous substances obtained by mixing beeswax with *Euphorbia* toxic sap [63]. Since the Neolithic Age, beeswax has also been used for the waterproofing of furniture, rituals, and cosmetics, and its use in ancient medicine dates back to ancient Egypt [60,64,65]. Over time, the usage of beeswax has been documented in sculpture, ornaments, masks, and candles, and at present this substance is exploited for the production of comb foundations in beekeeping, but also in the food industry as a glazing agent in fruits. For example, in the European Union, beeswax is an authorised food additive (E901) (EU Commission Regulation No. 1147/2012, 4 December 2012). 

Food packaging made from beeswax and other natural substances has been recently developed [66] and studies on the use of beeswax as a gelling agent in some food products are on-going [67].

Comprehensive reviews on the medicinal and pharmaceutical use of beeswax are available [60,68]. Beeswax has antimicrobial and antifungal activities and wax extracts possess antioxidant properties [60,69]. In pharmaceutical preparations, beeswax may act as a thickener, binder, drug carrier, and release retardant [70,71] and in surgery, as a mechanical barrier to control bleeding [72]. This substance is also widely used as a raw material in the modern cosmetic industry, being a common ingredient of lipsticks, sticks, and cream [73,74]. In addition, the use of beeswax as a biodegradable, not toxic substance in release-controlled pesticide formulations is also gaining attention [75].

Finally, the peculiar mechanical and thermal characteristics of this substance have also attracted the attention of engineers, who are promoting studies on applied energy systems [76]. Indeed, beeswax is characterised by a latent heat of 141.49 kJ/kg and a melting point of 62.28°C and the role of beeswax as a starting renewable raw material for thermal energy storage is promising [77]. Beeswax exhibits excellent potential for use as a phase-change material to decrease the battery temperature in electric vehicles [78]. In addition, beeswax may promote the mechanical properties of concrete [79].

### 3.2. Propolis

Propolis, commonly known as the ‘bee glue’, is a resinous substance that bees collect from plants and trees, buds, and exudates of plants, which are transformed in the presence of bee enzymes. Bees use propolis for the construction and adaptation of their nests, seal the holes in their honeycombs, smooth out internal walls, and cover carcasses of intruders who died inside the hive in order to avoid their decomposition [80]. Propolis also protects the colony from diseases because of its antiseptic and antimicrobial properties. 

The use of propolis has a long history and goes back to ancient times, as a local medicine in many parts of the world. Egyptians, Greeks, and Romans reported the use of propolis for its general healing qualities and for the cure of skin problems [81]. Propolis has always been used as an anti-inflammatory agent and to heal sores, ulcers, wounds, and for tissue regeneration [82]. 

In general, propolis is composed of 30% wax, 50% resin and vegetable balsam, 10% essential and aromatic oils, 5% pollen, and other components [83,84]. Its chemical composition is very complex: more than 300 components have already been identified, and its composition is dependent on the vegetal source and the local flora (geographical origin), thus creating a problem for its medical use and standardisation [84,85,86]. The main components are phenolic compounds (flavonoids, aromatic acids, and benzopyranes), di- and triterpenes, and essential oils, among others [87,88,89].

The antimicrobial properties and activities of propolis have been widely investigated [90,91,92,93,94]. Propolis also shows antiviral [95,96], antifungal [97,98], and antiparasitic activities [99,100,101].

Owing to its properties, propolis is used in products for the protection of health and prevention of diseases, in bio-pharmaceuticals, and as a constituent of bio-cosmetics [102,103]. Propolis-based products are also marketed by the pharmaceutical industry and health-food supply chains [104]. However, further investigations are needed to better understand the effects of propolis on human health and to establish its potential dose levels and intake periods. The recent systematic review and meta-analysis of randomised controlled clinical trials of Gheflati and colleagues [105] on the effects of propolis supplementation on metabolic parameters is worth mentioning. The current meta-analysis revealed that propolis supplementation can reduce aspartate aminotransferase.

Emerging directions are also given by the application of nanotechnologies to nutraceuticals and pharmaceuticals [106,107]. For instance, Botteon et al. [108] described the biosynthesis and characterisation of gold nanoparticles using Brazilian red propolis and evaluated their antimicrobial and anticancer activities. 

In the area of functional food, Cedeño-Pinos et al. [109] described the contribution of green propolis to produce more stable and healthier fruity jelly candies made with sugars or fructans. Rodrigues et al. [110] developed propolis co-product extracts as a natural antioxidant to reduce lipid oxidation in fatty starch biscuits of Brazil.

Another interesting example of the application of propolis is its use as a biopreservative for fruit juices [111].

### 3.3. Royal Jelly

Royal jelly is a secretion of the mandibular and hypopharyngeal glands of worker bees, *A. mellifera*. It is the food that regulates the distinction between reproductive and unreproductive females; only larvae exclusively fed on royal jelly develop into queens; otherwise, they develop into sterile workers [112]. 

Royal jelly is composed of 60–70% water, 9–18% protein, 7–18% simple sugars (monosaccharides), and 3–8% lipids [113]. It also contains trace minerals, pantothenic acid (vitamin B5), pyridoxine (vitamin B6), trace amounts of vitamin C, nucleotides, heterocyclic compounds, 10-hydroxy-2-decenoic acid (10-HDA), amino acids, and others [114]. Concerning the protein content, the major royal jelly proteins (MRJPs) [115] are a family of proteins secreted by the honeybees. Royal jelly has been used in traditional medicine since ancient times, and MRJPs are believed to be the main medicinal components. Other components include 10-acetoxydecanoic acid, trans-10-acetoxydec-2-enoic acid, 11- oxododecanoic acid, (11S)-hydroxydodecanoic acid, (10R,11R)-dihydroxydodecanoic acid, 3,11-dihydroxydodecanoic acid, and (11S),12-dihydroxydodecanoic acid [116]. 

Royal jelly has been widely used in commercial medical products, health foods, and cosmetics in many countries for more than 30 years [117]. A recent review by Guo et al. [118] summarised the biologically active role of royal jelly in the maintenance of biological functions, such as immunity, lifespan, memory, digestive system, blood glucose, obesity, antibacterial, and anti-cancer properties [116]. Ahmad et al. [114] provided new insights into the biological and pharmaceutical properties, such as antimicrobial, antioxidant, wound healing, immunomodulatory, anti-aging, anti-cancer, anti-inflammatory, anti-hypertension, anti-hyperlipidemic, oestrogenic, and neurotrophic effects of royal jelly.

Despite the numerous studies dedicated to royal jelly, there is a challenge for future research on this topic, including clinical trials with human participants to record the health benefits after regular consumption of this substance and set of intake limits to achieve this goal.

### 3.4. Venom and Apitherapy

In the eusocial Aculeate Hymenoptera, the venom and stinging apparatus initially evolved as devices to immobilise prey, and then became weapons to defend the colony mainly from the attacks of invertebrate and vertebrate predators [119]. In particular, honey bee colonies are rewarding targets for predators and hunters because of the rich storage of honey and pollen, and the mass of immature broods and adults [119].

*A. mellifera* venom is a valuable product harvested from honeybees, with a price ranging between $30 and $300 per gram. However, bee venom is a marginal product of apiculture [120,121].

Bee venom is a natural toxin secreted from a specific venom gland located in the bee abdomen and is injected through the sting. Bee venom consists of simple organic molecules, peptides, proteins, and other bioactive elements [119,122]. In particular, bee venom contains polypeptides such as melittin, apamin, and mast cell degranulating peptides, amines, such as histamine, serotonin, dopamine, and norepinephrine, and enzymes, such as phospholipase, hyaluronidase, and histidine decarboxylase [120]. Melittin is a basic 26-amino-acid polypeptide that is the main component of *A. mellifera* venom and represents 40–60% of dry venom [123,124]. Melittin has several toxicological, pharmacological, and biological effects, such as haemolysin activity, antibacterial, and antifungal activities, anti-tumour properties, and intense surface activity on cell lipid membranes [123,125,126]. Nevertheless, ecological factors (temperature, flowering stage, and site) can influence the composition and diversity of the peptide and the weight of the bee venom [120].

Bee venom has been used in therapeutic applications in oriental traditional medicine since 1000–3000 BCE for treating inflammatory diseases and pain [127]. Recently, several studies have proposed bee venom as a promising neuroprotective therapy for Parkinson’s disease and as an effective treatment for patients with multiple sclerosis and other autoimmune diseases, such as rheumatoid arthritis [128,129,130].

Long thought to be primarily linked to health treatments with bee venom, apitherapy is now recognised as a type of complementary medicine that uses different honeybee products, including honey, pollen, propolis, royal jelly, and api-air (i.e., inhalation of air from the hive), to prevent and/or treat health disorders [131,132].. 

Since ancient times, the use of bee products has been found in China, Korea, Egypt, Russian, and Greece traditional medicine practices [133] and apitherapy is currently used in traditional medicine in Africa, Europe, Asia, and South America [134].

Apitherapy is increasingly becoming the basis of api-tourism, i.e., a kind of tourism focused on physical health and well-being [135,136]. Kotova and Lesnikov [137] discussed and marked the symbiosis of apitherapy and tourist-recreational resources in health tourism as a factor of effective post-COVID-19 health. 

Apitherapy is also promoted as an alternative medicine, but its health claims are not always supported by sufficient scientific evidence [138]. For example, bee venom or other honeybee products were ineffective for the treatment or prevention of cancer, as reported by Russell and Rovere [139]. Adverse reactions to bee venom therapy are also frequent [140]. Regular exposure to the venom can also lead to arthropathy [141]. In sensitised persons, venom compounds can act as allergens, causing a wide range of allergic reactions, ranging from mild, local swelling to severe systemic reactions, anaphylactic shock, or in extreme cases, even in death [142]. From a future perspective, it is really an urge for researchers to conduct new clinical trials on the concept of apitherapy application to humans, to unveil its impact on health. It is worth investigating if potential health benefits can be obtained by regular consumption and treatment with these products. These questions can be addressed in the future and can provide consumers and patients with useful information.

## 4. Honey Bees and Bee Products to Safeguard Ecosystems from Pollution

A further role provided by honey bees is the possibility of delivering key information on the presence of pollutants in the environment. The first extensive study demonstrating that *A. mellifera* is an effective biological monitor of environmental contaminants over large geographic areas dates back to the 1980s [143].

This notable role is due to the morphological and behavioural characteristics of bees that, during their wide-ranging foraging activity, are highly exposed to organic and inorganic pollutants contaminating air, water, soil, and vegetation. Pollutants can also contaminate the bee products, such as pollen, honey, wax, propolis, and royal jelly. 

The use of honey bees provides the following advantages over other pollution monitoring systems:

1. Very limited purchase costs and maintenance—beekeeping is an easy and low-cost activity, which provides a potentially unlimited supply of bioindicators in many environments;
2. Self-sustaining biosensors for the pollutant collection;
3. Reliable samplers of pollutants, as the bees can fly for more than 3 km around a barycentre (the hive), exploring flowers, vegetation, water, and air for a maximum of three weeks.
4. No environmental impact.
5. Simultaneous collection of a wide range of pollutants during the foraging behaviour;
6. Collection of evidence for pollutants to enter the food chain (e.g., through honey or other edible bee products) and to expose pollinators to pollutant ingestion.

In addition, as a living organism, the bee also offers the option to study lethal and sublethal effects of pollutant exposure on a biological system. 

In the following paragraph, an overview of studies involving pollution in honey bees and bee products is provided.

### Pollution in Bees and Bee Products

Biomonitors or bioindicators include organisms that provide information on the quality of the environment [144]. Importantly, a biomonitor is always a bioindicator, whereas a bioindicator does not necessarily meet the requirements of a biomonitor [144]. 

Bioindicators are used to assess both health and changes in the environment they inhabit [145]. Three types of bioindicators can be distinguished: plants (e.g., diatoms and lichens), animals (e.g., aquatic invertebrates), and microbes. Bioindicators can be further distinguished into four categories depending on the application, namely ecological, environmental, biodiversity, and pollution bioindicators [145]. *A. mellifera* represents, together with its products, the most complete biosensor (bioindicator and bioaccumulator), which can provide a considerable amount of data on the state of health of the environment [146,147,148,149,150,151,152,153,154,155]. Each forager bee manages to cover a foraging distance of more than 3 km from the hive and, in some cases, the area covered can be up to 100 km^2^ [156,157]. While passing from flower to flower, it comes into contact with a large number of pollutants. 

Honeybees may accumulate pollutants in many ways. During flight and foraging activities, they collect airborne particulate matter and dust deposit on the surfaces on which the bee lands [158,159,160,161]. In addition, bees are exposed to pollutants through water used for both drinking and cooling the hive or to pollutants absorbed by the plants from the soil and accumulated in pollen and nectar [162]. 

Pollutants collected by bees can accumulate in honey, wax, pollen bullets, propolis, or other products (bees as collectors). Contaminants may also concentrate on the body of larvae or adults (bees as accumulators) [163,164]. 

The validity of the bee as a biological indicator has been demonstrated for the following pollutants:

- Agrochemicals
- Heavy metals
- Polycyclic aromatic hydrocarbons
- Radionuclides
- Dioxin, polychlorinated biphenyls,
- Particulate matter

In rural areas poor in wild vegetation, a bee is an excellent bioindicator of phytosanitary products; in this case, the insect is obliged to forage on or near cultivated species and will therefore come into contact with any sprayed active substance [165]. In these situations, a bee becomes a valid tool to identify times and ways of using substances at risk of toxicity and highlights the possible improper use of pesticides in real time [166].

Compared to rural areas, large urban agglomerations may be contaminated with pollutants from vehicular exhausts, which, in turn, may pollute the nectar and honeydew, the raw material for honey production [167]. Honey may thus contain pollutants that are characteristic of the environment [168], including minerals of natural or anthropogenic origin [169]. Exhaust fumes from vehicles may also interfere with the scents that ‘drive’ bees to the flowers they feed on [170]. Indeed, bees can perceive flower scents up to 1200 m away, although, due to pollution, this capacity reduces to 200–300 m. 

Several studies have used bee products, such as honey and pollen, as biomonitors of heavy metals, isotopic lead, and radioactivity [160,163,171,172,173,174,175]. Studies on trace element concentrations and lead isotopic compositions of honey reflect proximity to anthropogenic activities, such as shipping ports and heavy traffic [174,176]. Lead, originating mainly from motor traffic, can contaminate air, thus directly contaminating nectar and honeydew [177]. Lead and cadmium are considered the main toxic heavy metals and are thus the most frequently studied. Generally, lead is not transported through plants. On the other hand, cadmium originating from the metal industry and incinerators is transported from the soil to plants and can then contaminate nectar and honeydew. However, according to some studies, bees may also act as ‘biofilters’ that prevent elements from penetrating the bee products [178]. As reported by Bogdanov [179], metal contamination levels are lower in honey than in the bees, indicating that bees can filter and purify nectar to remove these contaminants. Similarly, Roman et al. [168], Ruschioni et al. [180], Saunier et al. [173], Losfeld et al. [181], and Conti et al. [182] observed that bees could partially purify nectar from heavy metals during honey production. Therefore, honey produced in areas characterised by high environmental contamination with heavy metals may be within the residue limits for contamination with elements/minerals [183].

In addition, a bee returning to the hive allows, through chemical analysis and numerical control of the population, one to identify any pollutants spread even in areas far from the station. For example, insecticides, such as phosphorganics and carbamates, cause neurotoxic effects, resulting in rapid death [184]. The number of dead bees is directly proportional to the toxicity and danger of the active ingredient used. Thus, the absence of bees in a biotope highlights the existence of unfavourable conditions due to the absence of food sources (e.g., intensive monocultures of plants with non-entomophilous pollination and exclusion of weeds) or to the presence of contaminants with a high toxicological risk for the insect. The death of bees is an increasingly serious problem worldwide. Colony collapse disorder, a phenomenon responsible for the huge loss of colonies in the USA, has been linked to many causes, including the sublethal effects of exposure to pollutants [185].

## 5. Cultural Ecosystem Services and Bees

Cultural ecosystem services are the nonmaterial benefits people obtain from the ecosystem through spiritual enrichment, cognitive development, reflection, recreation, and aesthetics [1]. The ecosystem and its components, processes, and diversity provide the basis for education in many societies, influencing the types of social relations (e.g., agricultural societies differ in many respects from nomadic herding societies) and the diversity of cultures [1].

**Figure 2:**
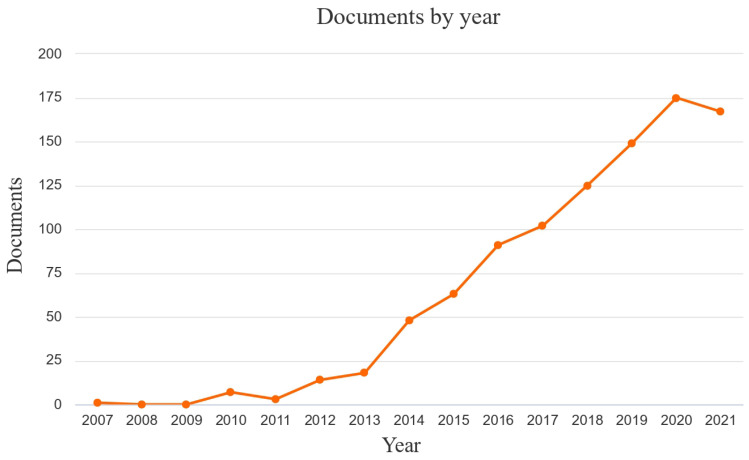
Scopus trend of the documents on cultural ecosystem services from 2007 to 2021 (Data from Scopus online Database; accessed on 8 November 2021).

A brief analysis of Scopus of all the available literature from 2007 to 2021 (accessed 8/11/2021) can help understand the impact of cultural ecosystem services on our community. From 2007 to 2021, we have 963 documents mentioning ‘cultural ecosystem services’, either in the title, keyword, or abstract. One single document was found in 2007, whereas in the last five years, the annual number of documents exceeded 100, indicating a growing interest in this topic (Figure 2).

Besides provisioning and regulating ecosystem services, honey bees and beekeeping can also be linked to cultural services. Among them, the best-known examples are recreational/educational activities, such as api-tourism, a form of agro-tourism related to beekeeping, which includes a number of beekeeping-oriented activities (e.g., visits to apiaries, bee museums, honey tasting, and apitherapy), and art inspiration [135,186,187].

Here, we will focus on the role of the honey bee in symbolic tradition and mysticism and the cultural values of the pollinator habitats.

### 5.1. The Role of the Honey Bee in the Ecosystem of the Symbolic Tradition

Among the infinite hermeneutic streams of essays, studies, and interpretations of the Bible, there is no shortage of specific works on the role and meaning of honey bees [188]. Heirs of the medieval bestiaries, the modern dictionaries of symbols [189,190], at the entry ‘bee’, always carry a rich collection of passages, legends, and myths about this insect and its mystic role. 

The highlights converge: in all cultural systems (from China to India, from Africa to Europe, from Babylonians to Christians), the honey bee has been respected, loved, and admired. This unanimity is noticeable: animals are usually ambiguous and have opposite meanings, even in the same cultural tradition. A lion at the portal of a Gothic cathedral represents the major power of redemption, but the same animal, holding a man in its paws, can signify the tremendous threat of sin. The positiveness of the bee was granted by generations of wisemen, who observed the presence of the bee in nature. They couldn’t keep from being seduced not only by the divine features of her products, but also by the dancing beauty of her trajectories. The symbolism always revolves around three main ambits: the sweetness of honey (and the pureness of wax), the organisation of the hive, and the nobleness of the queen bee. 

It is easy to understand how honey was considered important and almost sacred in ancient times. Source of sweetness, medicines, and gods liquor (the legendary hydromel), unique to the golden transparency of its flow, even at the dawn of history, it was gathered in the wilderness, before the introduction of beekeeping. In the history of Israel, the Promised Land is presented by God as a «land of milk and honey» (Exodus 3:8) and John the Baptist ate «locusts and wild honey» (Matthew 3:4). However, for the Bible, bees—as all insects—are impure animals, honey is kasher: it is the miracle of a sacrality produced by profane beings, such as humans. The fascinating work of the bees, who draw the ingredients from the fragility of flowers without spoiling them, has been a symbol of chastity, a virtue which was considered in a wider sense than mere sexuality: the sweetest things, in life, are obtained without grab, through the lightness of a caress. Thanks to modern science, we now know that life itself, through pollination, is granted by that caress.

The organisation of the hive, the laboriousness of the community, and the ability to work in the summer to ensure provisions for the winter, have often represented a perfect image of the ideal human society. The Church has observed in the storage of honey the treasure of good actions accumulated by the saints for the Heavenly Kingdom; the secular institutions have dreamed of the same ability to cooperate in such a harmonic way, even in large numbers. It is not surprising that bees and beehives have played an important role in the Western heraldry, carved on the shields of kings, nobles, and even monasteries, as in the coat of arms of the Cistercian abbey of Mellaray, which owes its name to honey (miel). 

Finally, the queen bee’s majesty has provided a sense of unity and obedience, a perfect representation of the monarchic government (in which a single head guarantees peace for the whole community), but also an image of the Mother Church, who is responsible for the destiny of every single believer. In the Catholic Exsultet, a long chant delivered before the paschal candle during the Easter night, the ‘mother bee’ is thanked for having produced the wax that makes the celebration possible, enlightening the dark heart of the night. All these features are well known, and the comprehension of the symbolism of the honeybee through simple bibliographic research can be easily deduced. 

Dictionaries and essays, however, usually do not show the complex dynamism of every symbolic process. A symbol is not only an arbitrary connection between a signifier (e.g., the honey bee) and a signified (obedience, pureness, and nobility): «the symboliser and the symbolised do not link together by chance’. Belyi-Florenskij quoted ‘we cannot invent symbols: they come themselves when you are filled with other content» [191]. The symbolic world arises from careful observation, daily relationships with the living natural reality, and acute sensitivity for the world. Once the modern man weakened his bonds with the natural world, the symbols became less significant. Once symbols started to lose power, the encounter with nature became merely instrumental. The last step of this process is the loss of many fundamental relationships, which used to be meaningful for humans. The majority of us, today, living in metropolitan areas, are deprived of those experiences which used to be fundamental in forging our symbolic rationality and behaviour: darkness, silence, the cycle of light, and of seasons. Natural reality has thus been transformed into a warehouse of materials, mute and meaningless, and therefore open to exploitation.

In 1967, Lynn White, in a famous and controversial article, accused Western culture to be responsible for that attitude toward nature, which is the root of today’s ecological crisis: having de-sacralised reality and having granted to man its monopoly—Christianity allowed the hubris of technology: «by destroying pagan animism, Christianity made it possible to exploit nature in a mood of indifference to the feelings of natural objects» [192]. The author, however, did not acknowledge that the symbolical process, which is the cornerstone of medieval culture, is an essential link between sacrality and mere exploitation. Symbols arise in an attitude of enchantment and wonder, which do not belong to religion alone, but also to art, literature, and poetry.

A symbolic system works in a similar way as an ecosystem: it is never a matter of a single element (the bee), but of the complex texture of relationships built by human imagination between all the elements that constitute our world. The purity of wax is explained by the darkness of the night, the sweetness of honey fulfils the strain of work, and the harmony of the hive interacts with the chaotic struggle of everyday life: water, earth, fire, and earth are the elements of the symphonic reality of symbols. Everything is connected: in symbols, as in nature. Isolating a symbol can provide some data, but it eventually hides the process of signification, and analysing the corpse of a bee under a microscope can provide important information, but it also implies the death of the insect. To deal with ecosystems, we must study the network of interactions; in order to understand symbols, we must dwell in the natural–cultural world and sharpen our sensitivity, because the symbolic universe awakens together with human perception. 

In this respect, there is one last feature of bees, which is particularly intriguing: they are often connected with language and words. The biblical word for bees, dəḇōrā (the name Deborah means honey bee) is derived from the root dbr, ‘word’: «Figuration de l’âme er du verbe—en hébreu le nom de l’abeille, Dbure, vient de la racine Dbr, parole—il est normal que l’abeille remplisse aussi un rôle initiatique et liturgique (Figuration of the soul and the verb—in Hebrew the name of the bee, Dbure, comes from the root Dbr, word—it is normal that the bee also fulfils an initiatory and liturgical role)» [189]. A famous medieval legend refers to St. Ambrose (his name comes from amber, thus similar to honey), as a sleeping baby, was visited by a swarm of bees, who poured into his mouth a mystical and mellifluous eloquence: «Ambrose, son of Ambrose the prefect of Rome, lay asleep in his cradle in the atrium of the palace when all of a sudden a swarm of bees flew in and covered his face and mouth so completely that the bees seemed to be moving in and out of their hive. Then they soared upward to such a height that the human eye could barely follow them» [193]. The bond which connects bees and words is meaningful: as in the ecosystem, bees maintain the role of pollination, in the cultural system words guarantee the generativeness of the discourse. The journey of pollen fertilises nature, and the journey of words generates culture. As the natural world would be doomed without bees, culture would disappear without the appropriate use of words. Mystical and yet prosaic, heavenly and yet hard working, coloured, yet humble, bees and words are the minimal elements of our reality. We do not know about the future, but we know that, if we want a future, it is our duty to protect them. I may appear as a dreamer, but I bet (all-in!) that the attitude to protect these little labourious insects and the attitude to protect the right use of our words can grow together: ecological commitment and poetry are the two Sisters’ Virtues. 

A sustainable behaviour, a ‘return to nature’, might not imply a new process of sacralisation, but it could benefit from the respect and restoration of human symbolic processes, which means a sense of care toward words. It is one of the ways we have to renovate our wonder towards nature, without falling into irrationality.

### 5.2. The Values of Pollinator Habitats

Habitats for pollinators are wildflower meadows rich not only in native flowering plants, but also in host plants and nesting/resting sites for a range of animals, from invertebrates to reptiles, birds, and small mammals [194,195]. Some amphibians also thrive on the food and shelter provided by the meadow ecosystem [196]. From an ecological perspective, pollinator habitats act as ‘keystone habitats’, which are extremely valuable habitats that provide critical resources to native organisms and are therefore a sanctuary for biodiversity [196,197]. 

With their blooms throughout the growing season, their scents and natural sounds, pollinator habitats are generally highly appreciated. They boost the aesthetics of rural landscapes [194]. Greening interventions in cities promoting wilderness and spontaneous flowering vegetation have become widely applied [198]. Indeed, flower meadows are easy to manage and offer an extraordinary opportunity to reconnect citizens with the wilderness within the urban environment, boosting the awareness that nature is not just a decoration but provides a living infrastructure as important as power grids and public transport [199].

Pollinator habitats in cities also provide a great opportunity to emphasise their educational value. Environmental education is considered a cultural ecosystem service, as it may provide appreciation for nature and natural areas as well as educational enrichment, especially in school children [200,201]. Outdoor learning in school is known to provide cognitive, physical, social-emotional, and academic benefits [202] and pollinator habitats in schoolyards may facilitate such outcomes. It may make children aware that biodiversity is always around us and «if we look around with curious eyes, we can see biodiversity not as colourful, not as exuberant as the tropical one, but not less important. In the end, a flowery meadow with wild chicory and chamomile is sufficient to appreciate the biodiversity. These plants are visited by many insects, pollinators, predators, and even herbivores, both in the larval and adult stages. You will need to get a sweep-net, swinging it with strength on the plants of this lawn. The insects living on these plants will be collected at the bottom of the bag. You will have to be a quick observer because insects try to regain freedom quickly or you can transfer everything you have collected with the net into a glass jar to observe them more calmly, obviously making sure you release all the insects once you have satisfied your curiosity. » (M. Pellecchia, [203]).

Given that the honey bee is undoubtedly the most known pollinator species and holds the public interest, it may be used as a flagship species, i.e., an icon for pollinator habitats. Thus, actions to support pollinator habitats may include the creation of sites for bees. However, to avoid negative impacts on wild pollinators due to competition of floral resources with *A. mellifera*, apiaries should be small and food resources (flowers) abundant [10]. 

**Figure 3:**
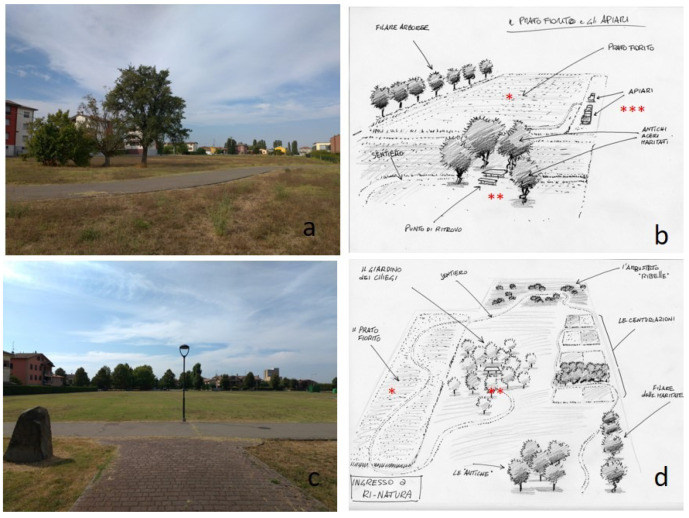
The Ri-natura project of the Sorbolo-Mezzani Municipality. Areas of intervention (**a**,**c**) and projects to transform them into multiservice green areas (**b**,**d**) with wildflower meadows (*), recreation/educational areas (**), and a small apiary (***). Photo courtesy of Sorbolo-Mezzani Municipality.

Many cases of cooperation among city councils and ecologists, zoologists, and entomologists to transform public green areas into open-air, living sanctuaries for pollinators, and other natural elements are available. One example is the ‘Ri-Natura’ project implemented in North Italy by the Sorbolo-Mezzani Municipality (Parma Province) to enhance citizens’ wellbeing and provide educational opportunities (Figure 3).

**Figure 4:**
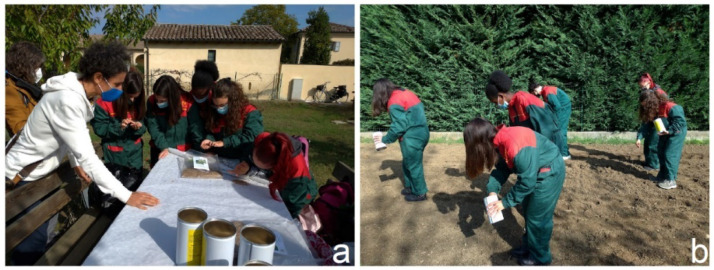
High school students at the Picasso Food Forest of Parma. (**a**) Volunteers providing knowledge to students on the importance of seeds of nectariferous and polliniferous wild plants; (**b**) students seeding the wildflower meadow (Erasmus Plus project, From Seed to Spoon 2019-1-IT02-KA201-062392).

Other examples of initiatives that improve well-being and nature education are urban food forests, i.e., small ecosystems planted with edible plants designed by humans to provide for their needs. Starting in 2010, food forests began to be included in municipal plans [204]. In many cases, food forests are also enriched by wildflower meadows to improve the presence of pollinators. One example is the Picasso Food Forest, in Parma, that, under the Erasmus Plus project From Seed to Spoon 2019-1-IT02-KA201-062392 involved high school students to plan, design, and seed a wildflower meadow to nourish pollinators [204] (Figure 4).

**Figure 5:**
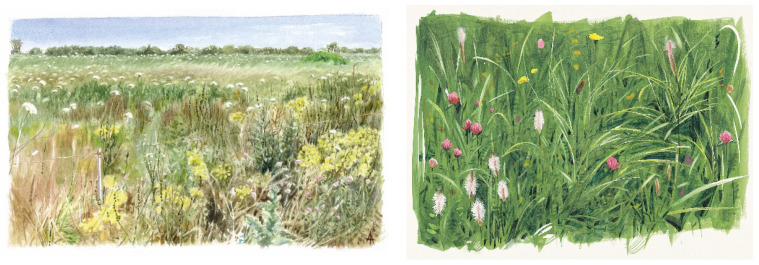
Pollinator habitats in naturalist paintings by A. Ambrogio. Courtesy of A. Ambrogio.

On the whole, the promotion of habitats for pollinators will, in turn, promote a range of cultural benefits. The educational, spiritual, recreational, and strongly inspiring value of nature is, in fact, a universally recognised value. Artists, who are well aware of this, often gain strength from the observation of natural beauty to produce their creations, and pollinator habitats are also frequently subject to naturalist illustrators (Figure 5).

Nature is the first inspiring muse of art. However, in reality, it is nature itself that is the great artwork.

## 6. Conclusions

Honey bees are the only insects that produce food for humans, as well as substances used as raw materials and pharmaceuticals. In natural ecosystems, this insect represents an important pollinator, thus contributing to the preservation of plant biodiversity, whereas pollination in agro-ecosystems can promote crop production. Furthermore, bees are key bioindicators of environmental pollution and may provide valuable information on the impact of human activities, enabling the implementation of measures to mitigate risks to human and ecosystem health.

The honey bee is also linked to many cultural ecosystem services, and has a longstanding tradition in human culture, mysticism, and religion. Its popularity may be used for educational purposes and to raise public awareness of important issues, such as conservation of pollinator habitats and biodiversity. Indeed, honey bees are a symbol of pollinators, widely recognised for their role in human and ecosystem health. We argue that the symbolic role of the bee is perhaps its most important role because it is just in the loss of the symbolic meaning that humans lose their intimate contact with nature. Now more than ever, at a time when modern society too often considers nature as a warehouse of materials, open to (over-)exploitation, the symbolic role of nature must be revitalised and reinforced. For all these reasons, we also believe that despite its usefulness in bringing policymakers and economists closer to environmental issues, the use of the concept of ‘Ecosystem Services’ is largely inaccurate, because it neglects the fact that humans are an integral part of ecosystems, humans are nature, and the role of humans in establishing and maintaining their social-ecological systems must not be overlooked.

## Figures and Tables

**Table 1:** Species list of known pollinators for global crops that are grown for direct human consumption [24,29].

Hymenoptera Pollination Groups	Species
Honey bee	*Apis cerana* Fabr., *A. dorsata* Fabr., *A. florea* Fabr. and *A. mellifera* L.
Stingless bees	*Melipona favosa* Fabr., *M. subnitida* Ducke, *M. quadrifasciata* Lepeletier, *Nanotrigona perilampoides* Cresson, *N. testaceicornis* Lepeletier, *Trigona cupira* Sm., *T. iridipennis* Smith, *T. (Lepidotrigona) terminata* Smith, *T. (Tetragonoula) minangkabau* Sakagami, *T. toracica* Smith and *Scaptotrigona depilis* Moure.
Bumble bees	*Bombus affinis* Cresson, *B. californicus* F. Smith, *B. hortorum* L., *B. hypnorum* L., *B. impatiens* Cresson, *B. lapidarius* L., *B. (Thoracobombus) pascuorum* Scop., *B. sonorus* L., *B. terrestris* L. and *B. vosnesenskii* Radoszkowski.
Solitary bees	*Amegilla chlorocyanea* Cockerell, *A. (Zonamegilla) holmesi* Rayment, *Andrena ilerda* Cam., *Anthophora pilipes* Fabr., *Centris tarsata* Smith, *Creightonella frontalis* Fabr., *Habropoda laboriosa* Fabr., *Halictus tripartitus* Cockerell, *Megachile (Delomegachile) addenda* Cresson, *M. rotundata* Fabr., *Osmia aglaia* Sandhouse, *O. cornifrons* Radoszkowski, *O. cornuta* Latreille, *O. lignaria lignaria* Say, *O. lignaria propinqua* Cresson, *O. ribifloris* Cockerell, *Peponapis limitaris* Cockerell, *P. pruinosa* Say, *Pithitis smaragdula* Fabr., *Xylocopa (Zonohirsuta) dejeanii* Lepeletier, *Xylocopa frontalis* Oliver and *Xylocopa suspecta* Moure.
Wasps	Fig wasps (e.g., *Blastophaga psenes* L.), *Tiphia vernalis* Rohwer

## Data Availability

Not applicable.

## References

[1] Island Press, Millennium Ecosystem Assessment (2005). Ecosystems and Human Well-Being: Synthesis.

[2] Fleming T.H., Muchhala N. (2008). Nectar-feeding bird and bat niches in two worlds: Pantropical comparisons of vertebrate pollination systems. J. Biogeogr..

[3] Ollerton J. (2017). Pollinator Diversity: Distribution, Ecological Function, and Conservation. Annu. Rev. Ecol. Evol. Syst..

[4] Ollerton J., Winfree R., Tarrant S. (2011). How many flowering plants are pollinated by animals?. Oikos.

[5] Potts S.G., Petanidou T., Roberts S., O’Toole C., Hulbert A., Willmer P. (2006). Plant-pollinator biodiversity and pollination services in a complex Mediterranean landscape. Biol. Conserv..

[6] Wardhaugh C.W., Stork N.E., Edwards W., Grimbacher P.S. (2012). The Overlooked Biodiversity of Flower-Visiting Invertebrates. PLoS ONE.

[7] Boyce C.K., Lee J.-E. (2010). An exceptional role for flowering plant physiology in the expansion of tropical rainforests and biodiversity. Proc. R. Soc. B Biol. Sci..

[8] Földesi R., Howlett B.G., Grass I., Batáry P. (2021). Larger pollinators deposit more pollen on stigmas across multiple plant species—A meta-analysis. J. Appl. Ecol..

[9] Garibaldi L.A., Steffan-Dewenter I., Winfree R., Aizen M.A., Bommarco R., Cunningham S.A., Kremen C., Carvalheiro L.G., Harder L.D., Afik O. (2013). Wild Pollinators Enhance Fruit Set of Crops Regardless of Honey Bee Abundance. Science.

[10] Iwasaki J.M., Hogendoorn K. (2021). How protection of honey bees can help and hinder bee conservation. Curr. Opin. Insect Sci..

[11] Requier F., Garnery L., Kohl P.L., Njovu H.K., Pirk C.W.W., Crewe R.M., Steffan-Dewenter I. (2019). The Conservation of Native Honey Bees Is Crucial. Trends Ecol. Evol..

[12] Hung K.-L.J., Kingston J.M., Lee A., Holway D.A., Kohn J.R. (2019). Non-native honey bees disproportionately dominate the most abundant floral resources in a biodiversity hotspot. Proc. R. Soc. B Biol. Sci..

[13] Henry M., Rodet G. (2020). The apiary influence range: A new paradigm for managing the cohabitation of honey bees and wild bee communities. Acta Oecologica.

[14] Fontana P., Costa C., Di Prisco G., Ruzzier E., Annoscia D., Battisti A., Caoduro G., Carpana E., Contessi A., Dal Lago A. (2018). Appeal for biodiversity protection of native honey bee subspecies of Apis mellifera in Italy (San Michele all’Adige declaration). Bull. Insectology.

[15] Hung K.-L.J., Kingston J.M., Albrecht M., Holway D.A., Kohn J.R. (2018). The worldwide importance of honey bees as pollinators in natural habitats. Proc. R. Soc. B Biol. Sci..

[16] Mallinger R.E., Gaines-Day H.R., Gratton C. (2017). Do managed bees have negative effects on wild bees?: A systematic review of the literature. PLoS ONE.

[17] Birks H.J.B., Felde V.A., Bjune A.E., Grytnes J.-A., Seppä H., Giesecke T. (2016). Does pollen-assemblage richness reflect floristic richness? A review of recent developments and future challenges. Rev. Palaeobot. Palynol..

[18] Galimberti A., De Mattia F., Bruni I., Scaccabarozzi D., Sandionigi A., Barbuto M., Casiraghi M., Labra M. (2014). A DNA Barcoding Approach to Characterize Pollen Collected by Honeybees. PLoS ONE.

[19] Prosser S.W.J., Hebert P.D.N. (2017). Rapid identification of the botanical and entomological sources of honey using DNA metabarcoding. Food Chem..

[20] Utzeri V.J., Schiavo G., Ribani A., Tinarelli S., Bertolini F., Bovo S., Fontanesi L. (2018). Entomological signatures in honey: An environmental DNA metabarcoding approach can disclose information on plant-sucking insects in agricultural and forest landscapes. Sci. Rep..

[21] Bell K.L., de Vere N., Keller A., Richardson R.T., Gous A., Burgess K.S., Brosi B.J. (2016). Pollen DNA barcoding: Current applications and future prospects. Genome.

[22] Milla L., Sniderman K., Lines R., Mousavi-Derazmahalleh M., Encinas-Viso F. (2021). Pollen DNA metabarcoding identifies regional provenance and high plant diversity in Australian honey. Ecol. Evol..

[23] Tremblay É.D., Duceppe M., Thurston G.B., Gagnon M., Côté M., Bilodeau G.J. (2019). High-resolution biomonitoring of plant pathogens and plant species using metabarcoding of pollen pellet contents collected from a honey bee hive. Environ. DNA.

[24] Klein A.M., Vaissière B.E., Cane J.H., Steffan-Dewenter I., Cunningham S.A., Kremen C., Tscharntke T. (2007). Importance of pollinators in changing landscapes for world crops. Proc. R. Soc. B Biol. Sci..

[25] Amaral G., Bushee J., Cordani U.G., Kawashita K., Reynolds J.H., Almeida F.F.M.D.E., de Almeida F.F.M., Hasui Y., de Brito Neves B.B., Fuck R.A., Intergovernmental Panel on Climate Change (2013). Summary for Policymakers. Climate Change 2013—The Physical Science Basis.

[26] McGregor S. (1976). Insect Pollination of Cultivated Crop Plants.

[27] Roubik D., FAO (1995). Services bulletin. Pollination of Cultivated Plants in the Tropics.

[28] Breeze T.D., Bailey A.P., Balcombe K.G., Potts S.G. (2011). Pollination services in the UK: How important are honeybees?. Agric. Ecosyst. Environ..

[29] Liu R., Chen D., Luo S., Xu S., Xu H., Shi X., Zou Y. (2020). Quantifying pollination efficiency of flower-visiting insects and its application in estimating pollination services for common buckwheat. Agric. Ecosyst. Environ..

[30] Rader R., Bartomeus I., Garibaldi L.A., Garratt M.P.D., Howlett B.G., Winfree R., Cunningham S.A., Mayfield M.M., Arthur A.D., Andersson G.K.S. (2016). Non-bee insects are important contributors to global crop pollination. Proc. Natl. Acad. Sci. USA.

[31] Doyle T., Hawkes W.L.S., Massy R., Powney G.D., Menz M.H.M., Wotton K.R. (2020). Pollination by hoverflies in the Anthropocene: Pollination by Hoverflies. Proc. R. Soc. B Biol. Sci..

[32] Cooley H., Vallejo-Marín M. (2021). Buzz-Pollinated Crops: A Global Review and Meta-analysis of the Effects of Supplemental Bee Pollination in Tomato. J. Econ. Entomol..

[33] Sáez A., Aizen M.A., Medici S., Viel M., Villalobos E., Negri P. (2020). Bees increase crop yield in an alleged pollinator-independent almond variety. Sci. Rep..

[34] Gonzalez M.V., Coque M., Herrero M. (1998). Influence of pollination systems on fruit set and fruit quality in kiwifruit (Actinidia deliciosa). Ann. Appl. Biol..

[35] Sagwe R.N., Peters M.K., Dubois T., Steffan-Dewenter I., Lattorff H.M.G. (2021). Pollinator supplementation mitigates pollination deficits in smallholder avocado (Persea americana Mill.) production systems in Kenya. Basic Appl. Ecol..

[36] Pardo A., Borges P.A.V. (2020). Worldwide importance of insect pollination in apple orchards: A review. Agric. Ecosyst. Environ..

[37] Hagen L. (2021). Pretty Healthy Food: How and When Aesthetics Enhance Perceived Healthiness. J. Mark..

[38] Pilati L., Fontana P., Angeli G. (2020). Commercial Pollination of Apple Orchards: Val di Non Case Study. Modern Beekeeping—Bases for Sustainable Production.

[39] Delaplane K., Mayer D. (2000). Crop Pollination by Bees.

[40] Hall M.A., Jones J., Rocchetti M., Wright D., Rader R. (2020). Bee Visitation and Fruit Quality in Berries Under Protected Cropping Vary Along the Length of Polytunnels. J. Econ. Entomol..

[41] Andrikopoulos C.J., Cane J.H. (2018). Comparative Pollination Efficacies of Five Bee Species on Raspberry. J. Econ. Entomol..

[42] Hünicken P.L., Morales C.L., Aizen M.A., Anderson G.K.S., García N., Garibaldi L.A. (2021). Insect pollination enhances yield stability in two pollinator-dependent crops. Agric. Ecosyst. Environ..

[43] Cunningham S.A., Fournier A., Neave M.J., Le Feuvre D. (2016). Improving spatial arrangement of honeybee colonies to avoid pollination shortfall and depressed fruit set. J. Appl. Ecol..

[44] Geslin B., Aizen M.A., Garcia N., Pereira A.-J., Vaissière B.E., Garibaldi L.A. (2017). The impact of honey bee colony quality on crop yield and farmers’ profit in apples and pears. Agric. Ecosyst. Environ..

[45] Grant K.J., DeVetter L., Melathopoulos A. (2021). Honey bee (*Apis mellifera*) colony strength and its effects on pollination and yield in highbush blueberries (*Vaccinium corymbosum*). PeerJ.

[46] Layek U., Kundu A., Bisui S., Karmakar P. (2021). Impact of managed stingless bee and western honey bee colonies on native pollinators and yield of watermelon: A comparative study. Ann. Agric. Sci..

[47] Bushmann S.L., Drummond F.A. (2020). Analysis of Pollination Services Provided by Wild and Managed Bees (Apoidea) in Wild Blueberry (Vaccinium angustifolium Aiton) Production in Maine, USA, with a Literature Review. Agronomy.

[48] Garibaldi L.A., Schulte L.A., Nabaes Jodar D.N., Gomez Carella D.S., Kremen C. (2021). Time to Integrate Pollinator Science into Soybean Production. Trends Ecol. Evol..

[49] Walters S.A., Taylor B.H. (2006). Effects of Honey Bee Pollination on Pumpkin Fruit and Seed Yield. HortScience.

[50] Cunningham S.A., Le Feuvre D. (2013). Significant yield benefits from honeybee pollination of faba bean (Vicia faba) assessed at field scale. Field Crops Res..

[51] Abbasi K.H., Jamal M., Ahmad S., Ghramh H.A., Khanum S., Khan K.A., Ullah M.A., Aljedani D.M., Zulfiqar B. (2021). Standardization of managed honey bee (Apis mellifera) hives for pollination of Sunflower (Helianthus annuus) crop. J. King Saud Univ.—Sci..

[52] Rizzardo R.A.G., Milfont M.O., da Silva E.M.S., Freitas B.M. (2012). Apis mellifera pollination improves agronomic productivity of anemophilous castor bean (*Ricinus communis*). An. Acad. Bras. Ciências.

[53] Romero M.J., Quezada-Euán J.J.G. (2013). Pollinators in biofuel agricultural systems: The diversity and performance of bees (*Hymenoptera: apoidea*) on Jatropha curcas in Mexico. Apidologie.

[54] Chauzat M.-P., Cauquil L., Roy L., Franco S., Hendrikx P., Ribière-Chabert M. (2013). Demographics of the European Apicultural Industry. PLoS ONE.

[55] Samarghandian S., Farkhondeh T., Samini F. (2017). Honey and Health: A Review of Recent Clinical Research. Pharmacogn. Res..

[56] Yilmaz A.C., Aygin D. (2020). Honey dressing in wound treatment: A systematic review. Complement. Ther. Med..

[57] Majtan J., Bucekova M., Kafantaris I., Szweda P., Hammer K., Mossialos D. (2021). Honey antibacterial activity: A neglected aspect of honey quality assurance as functional food. Trends Food Sci. Technol..

[58] Kostić A.Ž., Milinčić D.D., Barać M.B., Ali Shariati M., Tešić Ž.L., Pešić M.B. (2020). The Application of Pollen as a Functional Food and Feed Ingredient—The Present and Perspectives. Biomolecules.

[59] Svečnjak L., Chesson L.A., Gallina A., Maia M., Martinello M., Mutinelli F., Muz M.N., Nunes F.M., Saucy F., Tipple B.J. (2019). Standard methods for Apis mellifera beeswax research. J. Apic. Res..

[60] Fratini F., Cilia G., Turchi B., Felicioli A. (2016). Beeswax: A minireview of its antimicrobial activity and its application in medicine. Asian Pac. J. Trop. Med..

[61] Luo X., Dong Y., Gu C., Zhang X., Ma H. (2021). Processing Technologies for Bee Products: An Overview of Recent Developments and Perspectives. Front. Nutr..

[62] Sano K., Arrighi S., Stani C., Aureli D., Boschin F., Fiore I., Spagnolo V., Ricci S., Crezzini J., Boscato P. (2019). The earliest evidence for mechanically delivered projectile weapons in Europe. Nat. Ecol. Evol..

[63] La Nasa J., Nardella F., Andrei L., Giani M., Degano I., Colombini M.P., Ribechini E. (2020). Profiling of high molecular weight esters by flow injection analysis-high resolution mass spectrometry for the characterization of raw and archaeological beeswax and resinous substances. Talanta.

[64] Stacey R.J. (2011). The composition of some Roman medicines: Evidence for Pliny’s Punic wax?. Anal. Bioanal. Chem..

[65] Kramberger B., Berthold C., Spiteri C. (2021). Fifth millennium BC miniature ceramic bottles from the south-eastern Prealps and Central Balkans: A multi-disciplinary approach to study their content and function. J. Archaeol. Sci. Rep..

[66] Hao P., Lou X., Tang L., Wang F., Chong Z., Guo L. (2022). Solvent-free fabrication of slippery coatings from edible raw materials for reducing yogurt adhesion. Prog. Org. Coat..

[67] Sarkisyan V., Sobolev R., Frolova Y., Malinkin A., Makarenko M., Kochetkova A. (2021). Beeswax Fractions Used as Potential Oil Gelling Agents. J. Am. Oil Chem. Soc..

[68] Easton-Calabria A., Demary K.C., Oner N.J. (2019). Beyond Pollination: Honey Bees (*Apis mellifera*) as Zootherapy Keystone Species. Front. Ecol. Evol..

[69] Nainu F., Masyita A., Bahar M.A., Raihan M., Prova S.R., Mitra S., Emran T.B., Simal-Gandara J. (2021). Pharmaceutical Prospects of Bee Products: Special Focus on Anticancer, Antibacterial, Antiviral, and Antiparasitic Properties. Antibiotics.

[70] Cornara L., Biagi M., Xiao J., Burlando B. (2017). Therapeutic Properties of Bioactive Compounds from Different Honeybee Products. Front. Pharmacol..

[71] Ranjha N.M., Khan H., Naseem S. (2010). Encapsulation and characterization of controlled release flurbiprofen loaded microspheres using beeswax as an encapsulating agent. J. Mater. Sci. Mater. Med..

[72] Das J.M. (2018). Bone Wax in Neurosurgery: A Review. World Neurosurg..

[73] Kurek-Górecka A., Górecki M., Rzepecka-Stojko A., Balwierz R., Stojko J. (2020). Bee Products in Dermatology and Skin Care. Molecules.

[74] Prus-Walendziak W., Kozlowska J. (2021). Lyophilized Emulsions in the Form of 3D Porous Matrices as a Novel Material for Topical Application. Materials.

[75] Huang Y., Hu Q., Cui G., Guo X., Wei B., Gan C., Li W., Mo D., Lu R., Cui J. (2020). Release-controlled microcapsules of thiamethoxam encapsulated in beeswax and their application in field. J. Environ. Sci. Health Part B.

[76] Buchwald R., Breed M.D., Greenberg A.R. (2008). The thermal properties of beeswaxes: Unexpected findings. J. Exp. Biol..

[77] Baptista J.A., Eusébio M.E.S., Pereira M.M. (2021). New renewable raw materials for thermal energy storage. J. Therm. Anal. Calorim..

[78] Putra N., Sandi A.F., Ariantara B., Abdullah N., Indra Mahlia T.M. (2020). Performance of beeswax phase change material (PCM) and heat pipe as passive battery cooling system for electric vehicles. Case Stud. Therm. Eng..

[79] Umar H., Rizal S., Riza M., Mahlia T.M. (2018). Mechanical properties of concrete containing beeswax/dammar gum as phase change material for thermal energy storage. AIMS Energy.

[80] Damodaran T. (2021). Propolis. Nutraceuticals.

[81] Kuropatnicki A.K., Szliszka E., Krol W. (2013). Historical Aspects of Propolis Research in Modern Times. Evid.-Based Complement. Altern. Med..

[82] Ghisalberti E.L. (1979). Propolis: A Review. Bee World.

[83] Burdock G.A. (1998). Review of the biological properties and toxicity of bee propolis (propolis). Food Chem. Toxicol..

[84] Anjum S.I., Ullah A., Khan K.A., Attaullah M., Khan H., Ali H., Bashir M.A., Tahir M., Ansari M.J., Ghramh H.A. (2019). Composition and functional properties of propolis (bee glue): A review. Saudi J. Biol. Sci..

[85] Marcucci M.C. (1995). Propolis: Chemical composition, biological properties and therapeutic activity. Apidologie.

[86] de Castro S.L. (2001). Propolis: Biological and Pharmacological Activities. Therapeutic Uses of This Bee-product. Annu. Rev. Biomed. Sci..

[87] Boudourova-Krasteva G., Bankova V., Sforcin J.M., Nikolova N., Popov S. (1997). Phenolics from Brazilian Propolis. Z. Nat. C.

[88] Bankova V., Boudourova-Krasteva G., Popov S., Sforcin J.M., Funari S.R.C. (1998). Seasonal Variations in Essential Oil from Brazilian Propolis. J. Essent. Oil Res..

[89] Bankova V., Boudourova-Krasteva G., Popov S., Sforcin J.M., Cunha Funari S.R. (1998). Seasonal variations of the chemical composition of Brazilian propolis. Apidologie.

[90] Pasupuleti V.R., Sammugam L., Ramesh N., Gan S.H. (2017). Honey, Propolis, and Royal Jelly: A Comprehensive Review of Their Biological Actions and Health Benefits. Oxidative Med. Cell. Longev..

[91] Orsi R.O., Sforcin J.M., Funari S.R.C., Bankova V. (2005). Effects of Brazilian and Bulgarian propolis on bactericidal activity of macrophages against Salmonella Typhimurium. Int. Immunopharmacol..

[92] Orsi R.D.O., Sforcin J.M., Funari S.R.C., Fernandes Junior A., Bankova V. (2006). Synergistic effect of propolis and antibiotics on the Salmonella Typhi. Braz. J. Microbiol..

[93] Scazzocchio F., D’Auria F.D., Alessandrini D., Pantanella F. (2006). Multifactorial aspects of antimicrobial activity of propolis. Microbiol. Res..

[94] Nandre V.S., Bagade A.V., Kasote D.M., Lee J.H.J., Kodam K.M., Kulkarni M.V., Ahmad A. (2021). Antibacterial activity of Indian propolis and its lead compounds against multi-drug resistant clinical isolates. J. Herb. Med..

[95] Gekker G., Hu S., Spivak M., Lokensgard J.R., Peterson P.K. (2005). Anti-HIV-1 activity of propolis in CD4+ lymphocyte and microglial cell cultures. J. Ethnopharmacol..

[96] Liao N., Sun L., Wang D., Chen L., Wang J., Qi X., Zhang H., Tang M., Wu G., Chen J. (2021). Antiviral properties of propolis ethanol extract against norovirus and its application in fresh juices. LWT.

[97] Sforcin J.M., Fernandes Júnior A., Lopes C.A.M., Funari S.R.C., Bankova V. (2001). Seasonal effect of brazilian propolis on Candida albicans and Candida tropicalis. J. Venom. Anim. Toxins.

[98] Ibrahim M.E.E.-D., Alqurashi R.M. (2022). Anti-fungal and antioxidant properties of propolis (bee glue) extracts. Int. J. Food Microbiol..

[99] Salomao K., Dantas A.P., Borba C.M., Campos L.C., Machado D.G., Aquino Neto F.R., Castro S.L. (2004). Chemical composition and microbicidal activity of extracts from Brazilian and Bulgarian propolis. Lett. Appl. Microbiol..

[100] Freitas S.F., Shinohara L., Sforcin J.M., Guimarães S. (2006). In vitro effects of propolis on Giardia duodenalis trophozoites. Phytomedicine.

[101] Paula L.A., Cândido A.C.B.B., Santos M.F.C., Caffrey C.R., Bastos J.K., Ambrósio S.R., Magalhães L.G. (2021). Antiparasitic Properties of Propolis Extracts and Their Compounds. Chem. Biodivers..

[102] de Groot A.C. (2013). Propolis. Dermatitis.

[103] da Silva Barboza A., Aitken-Saavedra J.P., Ferreira M.L., Fábio Aranha A.M., Lund R.G. (2021). Are propolis extracts potential pharmacological agents in human oral health?—A scoping review and technology prospecting. J. Ethnopharmacol..

[104] Banskota A.H., Tezuka Y., Kadota S. (2001). Recent progress in pharmacological research of propolis. Phytother. Res..

[105] Gheflati A., Dehnavi Z., Ghannadzadeh Yazdi A., Khorasanchi Z., Raeisi-Dehkordi H., Ranjbar G. (2021). The effects of propolis supplementation on metabolic parameters: A systematic review and meta-analysis of randomized controlled clinical trials. Avicenna J. Phytomed..

[106] Souto E.B., Silva G.F., Dias-Ferreira J., Zielinska A., Ventura F., Durazzo A., Lucarini M., Novellino E., Santini A. (2020). Nanopharmaceutics: Part I—Clinical Trials Legislation and Good Manufacturing Practices (GMP) of Nanotherapeutics in the EU. Pharmaceutics.

[107] Souto E.B., Silva G.F., Dias-Ferreira J., Zielinska A., Ventura F., Durazzo A., Lucarini M., Novellino E., Santini A. (2020). Nanopharmaceutics: Part II—Production Scales and Clinically Compliant Production Methods. Nanomaterials.

[108] Botteon C.E.A., Silva L.B., Ccana-Ccapatinta G.V., Silva T.S., Ambrosio S.R., Veneziani R.C.S., Bastos J.K., Marcato P.D. (2021). Biosynthesis and characterization of gold nanoparticles using Brazilian red propolis and evaluation of its antimicrobial and anticancer activities. Sci. Rep..

[109] Cedeño-Pinos C., Marcucci M.C., Bañón S. (2021). Contribution of Green Propolis to the Antioxidant, Physical, and Sensory Properties of Fruity Jelly Candies Made with Sugars or Fructans. Foods.

[110] Rodrigues R., Bilibio D., Plata-Oviedo M.S.V., Pereira E.A., Mitterer-Daltoé M.L., Perin E.C., Carpes S.T. (2021). Microencapsulated and Lyophilized Propolis Co-Product Extract as Antioxidant Synthetic Replacer on Traditional Brazilian Starch Biscuit. Molecules.

[111] Chang Z.Q., Leong W., Chua L.S. (2021). Statistical approach to reveal propolis as a potential biopreservative for fruit juices. Future Foods.

[112] Kamakura M. (2011). Royalactin induces queen differentiation in honeybees. Nature.

[113] Melliou E., Chinou I. (2005). Chemistry and Bioactivities of Royal Jelly. J. Agric. Food. Chem..

[114] Ahmad S., Campos M.G., Fratini F., Altaye S.Z., Li J. (2020). New Insights into the Biological and Pharmaceutical Properties of Royal Jelly. Int. J. Mol. Sci..

[115] Uversky V.N., Albar A.H., Khan R.H., Redwan E.M. (2021). Multifunctionality and intrinsic disorder of royal jelly proteome. Proteomics.

[116] Melliou E., Chinou I. (2005). Chemistry and Bioactivity of Royal Jelly from Greece. J. Agric. Food Chem..

[117] Ramadan M.F., Al-Ghamdi A. (2012). Bioactive compounds and health-promoting properties of royal jelly: A review. J. Funct. Foods.

[118] Guo J., Wang Z., Chen Y., Cao J., Tian W., Ma B., Dong Y. (2021). Active components and biological functions of royal jelly. J. Funct. Foods.

[119] Baracchi D., Francese S., Turillazzi S. (2011). Beyond the antipredatory defence: Honey bee venom function as a component of social immunity. Toxicon.

[120] Scaccabarozzi D., Dods K., Le T.T., Gummer J.P.A., Lussu M., Milne L., Campbell T., Wafujian B.P., Priddis C. (2021). Factors driving the compositional diversity of *Apis mellifera* bee venom from a *Corymbia calophylla* (marri) ecosystem, Southwestern Australia. PLoS ONE.

[121] Hider R.C. (1988). Honeybee venom: A rich source of pharmacologically active peptides. Endeavour.

[122] de Lima P.R., Brochetto-Braga M.R. (2003). Hymenoptera venom review focusing on Apis mellifera. J. Venom. Anim. Toxins Incl. Trop. Dis..

[123] Chen J., Guan S.M., Sun W., Fu H. (2016). Melittin, the Major Pain-Producing Substance of Bee Venom. Neurosci. Bull..

[124] Silva J., Monge-Fuentes V., Gomes F., Lopes K., dos Anjos L., Campos G., Arenas C., Biolchi A., Gonçalves J., Galante P. (2015). Pharmacological alternatives for the treatment of neurodegenerative disorders: Wasp and bee venoms and their components as new neuroactive tools. Toxins.

[125] Son D., Lee J., Lee Y., Song H., Lee C., Hong J. (2007). Therapeutic application of anti-arthritis, pain-releasing, and anti-cancer effects of bee venom and its constituent compounds. Pharmacol. Ther..

[126] Park J.H., Jeong Y.-J., Park K.-K., Cho H.-J., Chung I.-K., Min K.-S., Kim M., Lee K.-G., Yeo J.-H., Park K.-K. (2010). Melittin suppresses PMA-induced tumor cell invasion by inhibiting NF-κB and AP-1-dependent MMP-9 expression. Mol. Cells.

[127] Zhang S., Liu Y., Ye Y., Wang X.R., Lin L.T., Xiao L.Y., Zhou P., Shi G.X., Liu C.Z. (2018). Bee venom therapy: Potential mechanisms and therapeutic applications. Toxicon.

[128] Khalil W.K.B., Assaf N., ElShebiney S.A., Salem N.A. (2015). Neuroprotective effects of bee venom acupuncture therapy against rotenone-induced oxidative stress and apoptosis. Neurochem. Int..

[129] Chung E.S., Kim H., Lee G., Park S., Kim H., Bae H. (2012). Neuro-protective effects of bee venom by suppression of neuroinflammatory responses in a mouse model of Parkinson’s disease: Role of regulatory T cells. Brain Behav. Immun..

[130] Mirshafiey A. (2007). Venom therapy in multiple sclerosis. Neuropharmacology.

[131] Cherbuliez T. (2013). Apitherapy—The Use of Honeybee Products. Biotherapy—History, Principles and Practice.

[132] Fratellone P.M., Tsimis F., Fratellone G. (2016). Apitherapy Products for Medicinal Use. J. Altern. Complement. Med..

[133] Mizrahi A., Lensky Y., Mizrahi A., Lensky Y. (1997). Bee Products. Properties, Applications, and Apitherapy.

[134] WHO (2019). WHO Global Atlas of Traditional, Complementary and Alternative Medicine.

[135] Topal E., Adamchuk L., Negri I., Kösoğlu M., Papa G., Dârjan M.S., Cornea-Cipcigan M., Mărgăoan R. (2021). Traces of Honeybees, Api-Tourism and Beekeeping: From Past to Present. Sustainability.

[136] Šuligoj M. (2021). Origins and development of apitherapy and apitourism. J. Apic. Res..

[137] Kotova T.P., Lesnikov A.I. (2021). Symbiosis of apiterapy and tourist—Recreational resources in health tourism as a factor of effective post-covidal health. Probl. Soc. Hyg. Public Health Hist. Med..

[138] Cassileth B.R. (2011). Apitherapy. The Complete Guide to Complementary Therapies in Cancer Care: Essential Information for Patients, Survivors and Health Professionals.

[139] Russell J., Rovere A. (2009). Apitherapy. American Cancer Society Complete Guide to Complementary and Alternative Cancer Therapies.

[140] Park J.H., Yim B.K., Lee J.-H., Lee S., Kim T.-H. (2015). Risk Associated with Bee Venom Therapy: A Systematic Review and Meta-Analysis. PLoS ONE.

[141] Cuende E., Fraguas J., Peña J.E., Peña F., García J.C., González M. (1999). Beekeeper’ arthropathy. J. Rheumatol..

[142] Vazquez-Revuelta P., Madrigal-Burgaleta R. (2018). Death due to live bee acupuncture apitherapy. J. Investig. Allergol. Clin. Immunol..

[143] Bromenshenk J.J., Carlson S.R., Simpson J.C., Thomas J.M. (1985). Pollution Monitoring of Puget Sound with Honey Bees. Science.

[144] Markert B.A., Breure A.M., Zechmeister H.G. (2003). Bioindicators and Biomonitors.

[145] Parmar T.K., Rawtani D., Agrawal Y.K. (2016). Bioindicators: The natural indicator of environmental pollution. Front. Life Sci..

[146] Lambert O., Piroux M., Puyo S., Thorin C., Larhantec M., Delbac F., Pouliquen H. (2012). Bees, honey and pollen as sentinels for lead environmental contamination. Environ. Pollut..

[147] Bargańska Ż., Ślebioda M., Namieśnik J. (2016). Honey bees and their products: Bioindicators of environmental contamination. Crit. Rev. Environ. Sci. Technol..

[148] Porrini C., Sabatini A.G., Girotti S., Ghini S., Medrzycki P., Grillenzoni F., Bortolotti L., Gattavecchia E., Celli G. (2003). Honey bees and bee products as monitors of the environmental contamination. Apiacta.

[149] Girotti S., Ghini S., Maiolini E., Bolelli L., Ferri E.N. (2013). Trace analysis of pollutants by use of honeybees, immunoassays, and chemiluminescence detection. Anal. Bioanal. Chem..

[150] Horn U., Helbig M., Molzahn D., Hentschel E.J. (1996). Transfer von 226 Ra in den Honig und die mögliche Nutzung der Honigbiene (Apis meilifera) als Bioindikator im radioaktiv belasteten Uranabbaugebiet der Wismut. Apidologie.

[151] Amorena M., Visciano P., Giacomelli A., Marinelli E., Sabatini A.G., Medrzycki P., Oddo L.P., De Pace F.M., Belligoli P., Di Serafino G. (2009). Monitoring of levels of polycyclic aromatic hydrocarbons in bees caught from beekeeping: Remark 1. Vet. Res. Commun..

[152] Mohr S., García-Bermejo Á., Herrero L., Gómara B., Costabeber I.H., González M.J. (2014). Levels of brominated flame retardants (BFRs) in honey samples from different geographic regions. Sci. Total Environ..

[153] Edo C., Fernández-Alba A.R., Vejsnæs F., van der Steen J.J.M., Fernández-Piñas F., Rosal R. (2021). Honeybees as active samplers for microplastics. Sci. Total Environ..

[154] Dively G.P., Kamel A. (2012). Insecticide Residues in Pollen and Nectar of a Cucurbit Crop and Their Potential Exposure to Pollinators. J. Agric. Food Chem..

[155] Martinello M., Manzinello C., Dainese N., Giuliato I., Gallina A., Mutinelli F. (2021). The Honey Bee: An Active Biosampler of Environmental Pollution and a Possible Warning Biomarker for Human Health. Appl. Sci..

[156] Perugini M., Manera M., Grotta L., Abete M.C., Tarasco R., Amorena M. (2011). Heavy Metal (Hg, Cr, Cd, and Pb) Contamination in Urban Areas and Wildlife Reserves: Honeybees as Bioindicators. Biol. Trace Elem. Res..

[157] Couvillon M.J., Ratnieks F.L.W. (2015). Environmental consultancy: Dancing bee bioindicators to evaluate landscape “health”. Front. Ecol. Evol..

[158] Negri I., Mavris C., Di Prisco G., Caprio E., Pellecchia M. (2015). Honey bees (*Apis mellifera*, L.) as active samplers of airborne particulate matter. PLoS ONE.

[159] Pellecchia M., Negri I. (2018). Particulate matter collection by honey bees (*Apis mellifera*, L.) near to a cement factory in Italy. PeerJ.

[160] Papa G., Capitani G., Capri E., Pellecchia M., Negri I. (2020). Vehicle-derived ultrafine particulate contaminating bees and bee products. Sci. Total Environ..

[161] Capitani G., Papa G., Pellecchia M., Negri I. (2021). Disentangling multiple PM emission sources in the Po Valley (Italy) using honey bees. Heliyon.

[162] Zaric N.M., Deljanin I., Ilijević K., Stanisavljević L., Ristić M., Gržetić I. (2018). Assessment of spatial and temporal variations in trace element concentrations using honeybees (*Apis mellifera*) as bioindicators. PeerJ.

[163] Leita L., Muhlbachova G., Cesco S., Barbattini R., Mondini C. (1996). Investigation of the use of honey bees and honey bee products to assess heavy metals contamination. Environ. Monit. Assess..

[164] González-Alcaraz M.N., Malheiro C., Cardoso D.N., Prodana M., Morgado R.G., van Gestel C.A.M., Loureiro S. (2020). Bioaccumulation and Toxicity of Organic Chemicals in Terrestrial Invertebrates.

[165] de Oliveira R.C., do Nascimento Queiroz S.C., da Luz C.F., Porto R.S., Rath S. (2016). Bee pollen as a bioindicator of environmental pesticide contamination. Chemosphere.

[166] Zioga E., Kelly R., White B., Stout J.C. (2020). Plant protection product residues in plant pollen and nectar: A review of current knowledge. Environ. Res..

[167] Mahé C., Jumarie C., Boily M. (2021). The countryside or the city: Which environment is better for the honeybee?. Environ. Res..

[168] Roman A., Majewska B., Pleban E. (2011). Comparative study of selected toxic elements in propolis and honey. J. Apic. Sci..

[169] Goretti E., Pallottini M., Rossi R., La Porta G., Gardi T., Cenci Goga B.T., Elia A.C., Galletti M., Moroni B., Petroselli C. (2020). Heavy metal bioaccumulation in honey bee matrix, an indicator to assess the contamination level in terrestrial environments. Environ. Pollut..

[170] Reitmayer C.M., Ryalls J.M.W., Farthing E., Jackson C.W., Girling R.D., Newman T.A. (2019). Acute exposure to diesel exhaust induces central nervous system stress and altered learning and memory in honey bees. Sci. Rep..

[171] Tonelli D., Gattavecchia E., Ghini S., Porrini C., Celli G., Botanico I. (1990). Honey bees and their products as indicators of environmental radioactive pollution a network of about 200 bee hives located far from the towns in different regions. J. Radioanal. Nucl. Chem..

[172] Satta A., Verdinelli M., Ruiu L., Buffa F., Salis S., Sassu A., Floris I. (2012). Combination of beehive matrices analysis and ant biodiversity to study heavy metal pollution impact in a post-mining area (Sardinia, Italy). Environ. Sci. Pollut. Res..

[173] Saunier J.B., Losfeld G., Freydier R., Grison C. (2013). Trace elements biomonitoring in a historical mining district (les Malines, France). Chemosphere.

[174] Smith K.E., Weis D., Amini M., Shiel A.E., Lai V.W.M., Gordon K. (2019). Honey as a biomonitor for a changing world. Nat. Sustain..

[175] Papa G., Capitani G., Pellecchia M., Negri I. (2021). Particulate Matter Contamination of Bee Pollen in an Industrial Area of the Po Valley (Italy). Appl. Sci..

[176] Zhou X., Taylor M.P., Davies P.J. (2018). Tracing natural and industrial contamination and lead isotopic compositions in an Australian native bee species. Environ. Pollut..

[177] Matin G., Kargar N., Buyukisik H.B. (2016). Bio-monitoring of cadmium, lead, arsenic and mercury in industrial districts of Izmir, Turkey by using honey bees, propolis and pine tree leaves. Ecol. Eng..

[178] Dżugan M., Dżugan M., Zaguła G., Wesołowska M., Sowa P., Puchalski C. (2017). Levels of toxic and essential metals in varietal honeys from Podkarpacie. J. Elem..

[179] Bogdanov S. (2006). Contaminants of bee products. Apidologie.

[180] Ruschioni S., Riolo P., Minuz R.L., Stefano M., Cannella M., Porrini C., Isidoro N. (2013). Biomonitoring with Honeybees of Heavy Metals and Pesticides in Nature Reserves of the Marche Region (Italy). Biol. Trace Elem. Res..

[181] Losfeld G., Saunier J.-B., Grison C. (2014). Minor and trace-elements in apiary products from a historical mining district (Les Malines, France). Food Chem..

[182] Conti M.E., Canepari S., Finoia M.G., Mele G., Astolfi M.L. (2018). Characterization of Italian multifloral honeys on the basis of their mineral content and some typical quality parameters. J. Food Compos. Anal..

[183] Erbilir F., Erdoĝrul Ö. (2005). Determination of Heavy Metals in Honey in Kahramanmaraş City, Turkey. Environ. Monit. Assess..

[184] Porrini C., Sabatini A., Girotti S., Fini F., Monaco L., Celli G., Bortolotti L., Ghini S. (2003). The death of honey bees and environmental pollution by pesticides: The honey bees as biological indicators. Bull. Insectology.

[185] Feldhaar H., Otti O. (2020). Pollutants and their interaction with diseases of social Hymenoptera. Insects.

[186] Aryal S., Ghosh S., Jung C. (2020). Ecosystem Services of Honey Bees; Regulating, Provisioning and Cultural Functions. J. Apic..

[187] Crane E. (2005). The rock art of honey hunters. Bee World.

[188] Carozza G. (2019). La parola è più dolce del miele. Le api e il miele nella Bibbia e nella tradizione cristiana.

[189] Chevalier J., Gheerbrant A. (1969). Dictionnaire des Symboles. Mythes, Rêves, Coutumes, Gestes, Formes, Figures, Couleurs, Nombres.

[190] Charbonneau-Lassay L. (1991). The Bestiary of Chris.

[191] Belyj A., Florenskij P., Giuliano G. (2004). L’ arte, il simbolo e Dio. Lettere sullo spirito russo.

[192] White L. (1967). The Historical Roots of Our Ecologic Crisis. Science.

[193] da Varagine J. (1993). The Golden Legend.

[194] Wratten S.D., Gillespie M., Decourtye A., Mader E., Desneux N. (2012). Pollinator habitat enhancement: Benefits to other ecosystem services. Agric. Ecosyst. Environ..

[195] Fleury P., Seres C., Dobremez L., Nettier B., Pauthenet Y. (2015). “Flowering Meadows”, a result-oriented agri-environmental measure: Technical and value changes in favour of biodiversity. Land Use Policy.

[196] Bonnet X., Brischoux F., Pearson D., Rivalan P. (2009). Beach rock as a keystone habitat for amphibious sea snakes. Environ. Conserv..

[197] Hitchman S.M., Mather M.E., Smith J.M., Fencl J.S. (2018). Identifying keystone habitats with a mosaic approach can improve biodiversity conservation in disturbed ecosystems. Glob. Change Biol..

[198] Sikorska D., Ciężkowski W., Babańczyk P., Chormański J., Sikorski P. (2021). Intended wilderness as a Nature-based Solution: Status, identification and management of urban spontaneous vegetation in cities. Urban For. Urban Green..

[199] Kowarik I. (2018). Urban wilderness: Supply, demand, and access. Urban For. Urban Green..

[200] Hutcheson W., Hoagland P., Jin D. (2018). Valuing environmental education as a cultural ecosystem service at Hudson River Park. Ecosyst. Serv..

[201] Mocior E., Kruse M. (2016). Educational values and services of ecosystems and landscapes—An overview. Ecol. Indic..

[202] van Dijk-Wesselius J.E., van den Berg A.E., Maas J., Hovinga D. (2020). Green Schoolyards as Outdoor Learning Environments: Barriers and Solutions as Experienced by Primary School Teachers. Front. Psychol..

[203] S2S—Erasmus Plus project From seed to spoon 2019-1-IT02-KA201-062392 Catching insect diversity with a sweep net 2020.

[204] Riolo F. (2019). The social and environmental value of public urban food forests: The case study of the Picasso Food Forest in Parma, Italy. Urban For. Urban Green..

